# Role of endocrine PACAP in age-related diseases

**DOI:** 10.3389/fendo.2023.1118927

**Published:** 2023-03-09

**Authors:** Denes Toth, Dora Reglodi, Lili Schwieters, Andrea Tamas

**Affiliations:** ^1^ Department of Forensic Medicine, University of Pécs Medical School, Pécs, Hungary; ^2^ Department of Anatomy, ELKH-PTE PACAP Research Team, Centre for Neuroscience, University of Pécs Medical School, Pécs, Hungary

**Keywords:** PACAP, aging, endocrine, diseases, body fluids, biomarker

## Abstract

Pituitary adenylate cyclase activating polypeptide (PACAP) is a conserved neuropeptide, which confers diverse anti-aging endocrine and paracrine/autocrine effects, including anti-apoptotic, anti-inflammatory and antioxidant action. The results of the *in vivo* and *in vitro* experiments show that increasing emphasis is being placed on the diagnostic/prognostic biomarker potential of this neuropeptide in a wide array of age-related diseases. After the initial findings regarding the presence and alteration of PACAP in different body fluids in physiological processes, an increasing number of studies have focused on the changes of its levels in various pathological conditions associated with advanced aging. Until 2016 – when the results of previous human studies were reviewed – a vast majority of the studies had dealt with age-related neurological diseases, like cerebrovascular and neurodegenerative diseases, multiple sclerosis, as well as some other common diseases in elderly such as migraine, traumatic brain injury and post-traumatic stress disorder, chronic hepatitis and nephrotic syndrome. The aim of this review is to summarize the old and the new results and highlight those ‘classical’ and emerging clinical fields in which PACAP may become subject to further investigation as a diagnostic and/or prognostic biomarker in age-related diseases.

## 1 Introduction

Rapid aging of the population is the leading health challenge in the Western world. It is expected that by mid-century the proportion of citizens over the age of 65 will reach over 30 percent in most states in the European Union (1). Similar trends are evident in Japan, the United States and many other developed countries (2, 3). Understanding and mitigating the biological processes underlying aging is essential to create healthier older populations. During the past several decades it has become evident that in addition to cell autonomous mechanisms of aging, cellular aging processes are also driven by signals originating from other cells. These cell non-autonomous pathways involve both endocrine and paracrine signaling mechanisms (4). The goal of this review is to provide an overview on the roles of an evolutionarily conserved neuropeptide, pituitary adenylate cyclase activating polypeptide (PACAP), in the development of age-related organ-specific pathologies.

**Figure 1:**
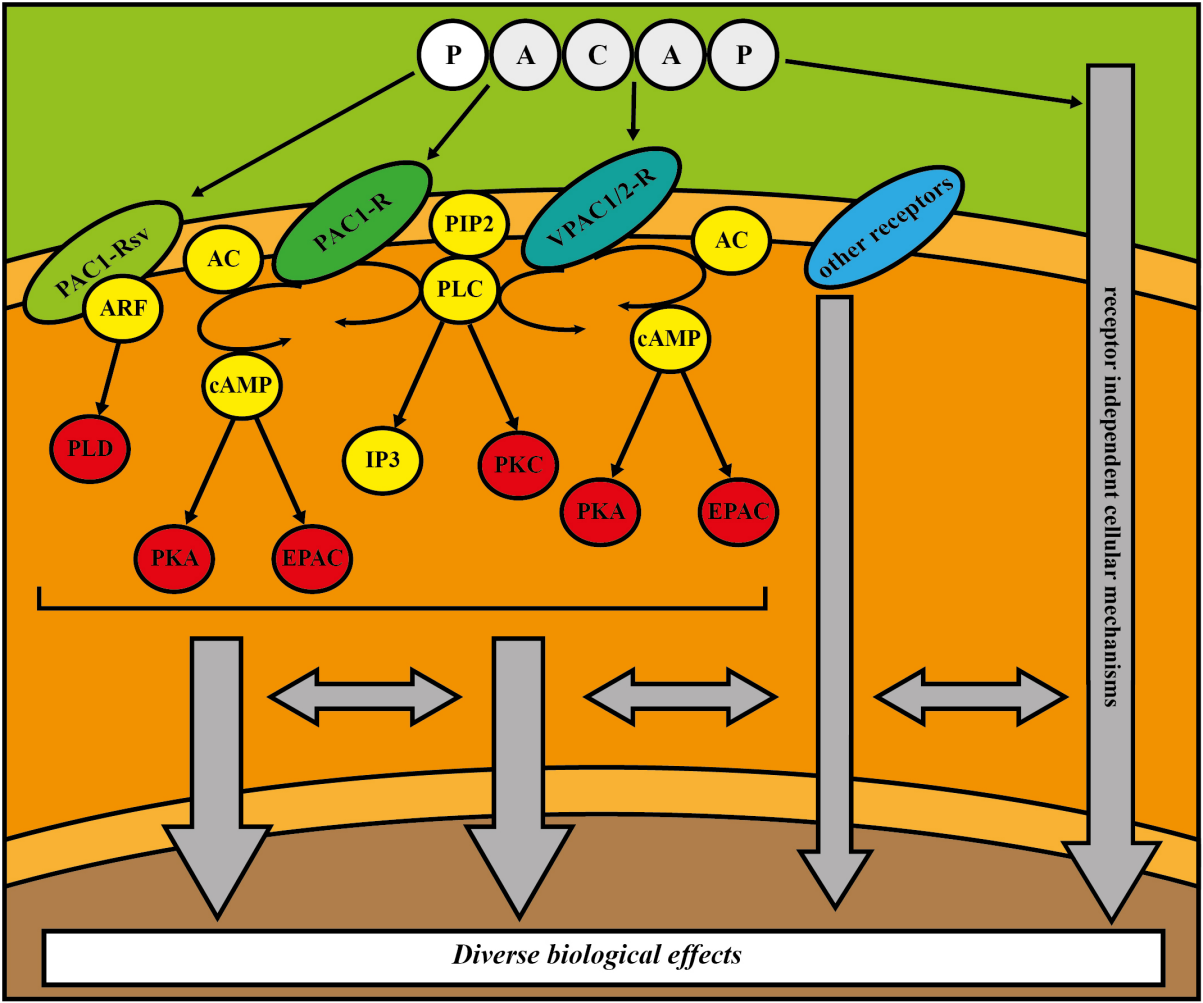
Schematic drawing of the possible signaling pathways regulated by PACAP. (AC = adenylate cyclase, ARF = adenosine diphosphate ribosylation factor, cAMP = cyclic adenosine monophosphate, EPAC = exchange proteins activated by cAMP, IP3 = 1,4,5–inositol trisphosphate, PACAP = pituitary adenylate cyclase activating polypeptide, PAC1–R = PACAP type I receptor, PAC1–Rsv = PACAP type I receptor splice variants, PIP2 = phosphatidylinositol bisphosphate, PKA = protein kinase A, PKC = protein kinase C, PLC = phospholipase C, PLD = phospholipase D, VPAC1/2–R = Pituitary adenylate cyclase–activating polypeptide type II receptor 1 or 2.

PACAP, a member of the secretin/glucagon/growth hormone-releasing hormone superfamily, is a multifunctional and pleiotropic neuropeptide with well-known anti-aging action, mediating anti-apoptotic, anti-inflammatory and antioxidant effects (5–11). There is increasing evidence that it plays important roles in preservation of youthful cellular phenotypes, maintenance of organ functions and modulation of the pathogenesis of age-related diseases. This evolutionarily highly conserved protein has two functionally active isoforms: PACAP38 (12) and PACAP27 (13). The residue containing 38 amino acids (PACAP38) is the predominant form in mammals, while PACAP27, which contains 27 amino acids, represents 10% of total PACAP in the body. PACAP and vasoactive intestinal peptide (VIP) share two G-protein-coupled receptors, namely VPAC1 and VPAC2, however the PAC1 receptor shows a 1000-fold increase in activity when bound to PACAP instead of VIP (14). PACAP receptors are coupled to G protein q or s, which stimulate the activation of adenylyl cyclase (AC) or phospholipase C (PLC). AC boosts adenosine triphosphate (ATP) conversion to cyclic adenosine monophosphate (cAMP), which then stimulates protein kinase A (PKA) phosphorylation and other cAMP downstream, such as exchange proteins activated by cAMP (EPAC). Activation of PLC increases both protein kinase C (PKC) and 1,4,5-inositol trisphosphate (IP3) function. Splice variants of PAC1 receptor and VPAC1 and 2 receptors can engage adenosine diphosphate ribosylation factor (ARF)- phospholipase D (PLD) pathway (6, 11, 14). The diverse biological effects of PACAP in the organs and tissues are determined by the expressed receptors and associated molecular pathways, receptor splice variants, transactivation of other receptors, transmembrane receptor independent cellular mechanisms, cell or tissue types, environmental factors, developmental stage, diurnal rhythm, the presence of noxious agents, and pathological conditions (6, 11, 14). The main signaling pathways are summarized in **Figure 1**.

Soon after its discovery, PACAP became one of the most extensively studied neuropeptides, as it was proven that PACAP and its receptors are broadly expressed in the nervous system, peripheral organs, as well as in body fluids (14). In line with the widespread distribution, PACAP has been found to be taking part in a variety of physiological processes ranging from reproduction (15) and development (16) to the pathogenesis of age-associated diseases (5–7). As the number of published articles increased exponentially, the high translational potential of PACAP became obvious. The description of its effects and localization in human tissues was followed by the detection of PACAP levels in various human body fluids. Publications on the alteration of PACAP levels in human physiological and pathological conditions associated with aging and age-related diseases were reviewed in 2016 (17). However, in the past 5 years new findings have broadened the realm of knowledge on this topic. The aim of this review is to summarize old and new results and highlight those ‘classical’ and emerging clinical fields in which PACAP may become subject to further investigation as a diagnostic and/or prognostic biomarker in age-related diseases.

## 2 Neurological and psychiatric diseases

In recent decades, new technological advances with increased precision and sensitivity have made it possible to detect structural and functional imaging biomarkers and fluid biomarkers as a measure of neuronal damage in a wide range of neurological conditions, such as neurodegenerative disorders, multiple sclerosis, traumatic brain injury or peripheral neuropathies (18). To better understand the role of cell non-autonomous mechanisms in the pathogenesis of these diseases in general and the specific role(s) of PACAP in particular the shift to a more systematic way of thinking is inevitable. Consequently, the mapping of potential candidates for further biomarker and therapeutic research could be possible (19).

The biomarker potential of PACAP was previously reviewed by Reglodi et al. involving spontaneous basal ganglia hemorrhage, acute non-traumatic aneurysmal subarachnoid hemorrhage, mild cognitive impairment preceding Alzheimer’s disease, Parkinson’s disease with dementia, frontotemporal lobar degeneration, children with intellectual disability of different etiologies, borderline IQ, migraine, tension type of headache, multiple sclerosis, post-traumatic stress disorder in females and traumatic brain injury (17).

### 2.1 Alzheimer’s disease

There is an increasing interest in understanding the role of fundamental mechanisms of aging in the pathogenesis of Alzheimer’s disease (AD) (20–22). In addition to cell autonomous mechanisms of aging there is growing evidence that cell non–autonomous mechanisms play a critical role in the development of amyloid pathologies and progression of the disease (23–25).

Han et al., in a case control study, examined the expression of PACAP messenger RNA and protein in brains of patients with pathologically confirmed late–onset AD and age–matched cognitively normal controls. Postmortem cisternal cerebrospinal fluid (CSF) samples were also examined. Higher cortical β–amyloid neuritic plaque density was correlated with lower PACAP expression in the entorhinal cortex and superior frontal gyrus but not the middle temporal gyrus or primary visual cortex. PACAP levels were inversely related to Braak stages V and VI compared with stages III and IV, suggesting that PACAP could reflect tau pathology. PACAP levels in the CSF were reduced in AD and correlated positively with Mattis Dementia Rating Scale–Revised scores, a measure of global cognitive functioning (26). Similar inverse correlation of PACAP with β–amyloid neuritic plaques and neurofibrillary tangles was found in another study by Han et al. (27). The results of these studies suggest that PACAP is not only reduced in AD, but also correlates with the severity of AD pathology. In a subsequent study, the same research group found that PACAP levels in CSF correlated positively with the Mattis Dementia Rating Scale score and negatively correlated with total amyloid plaques and tangles in the brain. PACAP levels in the superior frontal gyrus correlated positively with the Stroop Color–Word Interference Test and PACAP concentrations in the middle temporal gyrus correlated positively with the Auditory Verbal Learning Test–Total Learning scores. Furthermore, it was found that PAC1 expression in the superior frontal gyrus showed an upregulation in mild cognitive impairment due to AD but not in AD without dementia (28).

### 2.2 Parkinson’s disease

Parkinson’s disease (PD) is a prevalent age–associated neurodegenerative disease (29), the pathogenesis of which is modulated by both cell autonomous mechanisms and cell non–autonomous mechanisms of aging (30–32).

In PD, significantly lower serum PACAP levels were detected compared to those in healthy controls. Regarding motor symptoms severity, compared to PD patients with Hoehn–Yahr (H–Y) stages I–II, PACAP levels in H–Y stage III and in H–Y stages IV–V were lower, but there was no significant difference between these stages (33). Negative correlation was found between serum PACAP concentrations and disease duration. Receiver operating characteristic (ROC) curve analysis showed that PACAP had 74.6% sensitivity and 80.6% specificity at the cut–off value of 106.54 pg/mL. Regarding non–motor symptoms it was determined that PACAP levels were inversely correlated with only one element of the Non–motor Symptoms Scale for Parkinson’s disease, namely with attention/memory. Serum PACAP levels were lower in the cognitive dysfunction subgroup of PD than in the cognitively intact subgroup (33). Regarding cognitive dysfunctions, a previous study by Han et al. described no changes in CSF PACAP levels in Parkinson’s disease with dementia compared to cognitively normal controls (26).

A recent study examined plasma PACAP levels of PD patients and age–matched healthy controls focusing on clinical features including motor and non–motor symptoms and different therapeutical methods. Plasma PACAP levels were significantly lower in PD patients without deep brain stimulation (DBS) therapy compared to healthy controls, and DBS–treated patients had significantly higher plasma PACAP levels compared to PD patients who had not received DBS therapy. In samples from akinetic–rigid subtype had the lowest plasma PACAP levels, while the highest levels were detected in the mixed subtype. Regarding motor symptom severity, highest PACAP levels were found in the mildest, H–Y stage I group. A significant decrease of PACAP levels was detected until H–Y stage III. Significant differences were observed between H–Y stage II and III and between H–Y stage II and IV. Significant elevation of PACAP levels was observed in the most severe stage of Epworth sleepiness scale, measuring the severity of sleep disturbances. In PD patients older than 50 years at the time of diagnosis significantly lower PACAP levels were found compared to those who were younger, although patients without DBS treatment were older compared to DBS–treated patients (34). Interestingly, a similar age–related finding, as a transition decade, was described in case of human choroid plexus (CP) aging, which confirmed the results of previous *in vivo* experiments in which altered CP transcriptome was detectable in various central nervous system disorders (35).

### 2.3 Multiple sclerosis

The prevalence of multiple sclerosis (MS) has increased in every world region since 2013 and the average age of multiple sclerosis patients is rising. In 2008 MS prevalence peaked at age 55–59 years and approximately 14% of MS patients were aged 65 years and older in 2010. Aging is a significant factor influencing the course of MS (36).

In MS patients no significant difference was found in the median serum concentration of PACAP compared to healthy controls. In the MS group, however, significantly lower serum PACAP levels were found in males in contrast to females. No association was found between serum PACAP levels and MS disease type or history of previous relapses (37). These results are in accordance with an earlier study on this topic, in which the findings indicate that patients suffering from MS had decreased PACAP levels in CSF, while plasma levels of the polypeptide did not change (38).

### 2.4 Headaches

One of the most common neurological complaints of elderly patients is headache, and primary headaches comprise about two–thirds of headaches among the elderly. As the population ages, headaches in elderly are likely to become a more significant public health issue. The prevalence of chronic headaches, including chronic migraine, chronic tension type headaches, medication overuse headaches in the elderly ranges from 5 to 22% and occurs more frequently in women (39–41).

#### 2.4.1 Migraine

PACAP plays an essential role in the pathogenesis of primary headaches. Growing evidence supports the involvement of PACAPergic system in migraine and nowadays PACAP receptors becoming promising agents for migraine therapeutics. Beyond the therapeutical potential of PACAP, data from human studies also indicate PACAP to be a possible biomarker for migraines in the future (42).

In a study, significantly higher plasma PACAP levels were found in migraineur patients than in control subjects. Moreover, higher plasma PACAP concentrations were detected in migraineur patients in both the ictal and the interictal period than in the control group. However, there was no significant difference between ictal and interictal periods in plasma PACAP levels. In the interictal period, a negative correlation was detected between attack frequency per month and plasma PACAP levels (43). In contrast, earlier studies showed elevated plasma PACAP levels in ictal phase compared to the interictal period (44, 45).

Interictal plasma PACAP levels were examined in an exploratory study, where migraineur females underwent diffusion tensor imaging afterward. Interictal plasma PACAP levels showed significant correlation with mean diffusivity in the bilateral occipital white matter reaching into temporal and parietal white matter. The correlation with axial diffusivity was also significant in the left posterior corpus callosum and left optic radiation. Interictal PACAP levels correlated with radial diffusivity in the left parietal white matter and left optic radiation. With sex, age and disease duration – as nuisance regressors – interictal PACAP levels showed significant correlation with mean diffusivity and axial diffusivity in the left thalamus (46).

In another study, serum PACAP levels were measured in case of patients with chronic migraine, episodic migraine, and healthy controls without migraine attacks. There was no difference in chronic migraine patients compared to episodic migraine and healthy controls (47). In contrast with the pervious paper, in a cohort study, chronic migraine, episodic migraine and healthy controls were compared to assess the diagnostic value of PACAP and other neuropeptides in real clinical practice. Significantly higher serum PACAP levels were detected in chronic migraine patients than in the episodic migraine group and healthy controls. The observed ranges for PACAP were wider in chronic migraine than episodic migraine and healthy controls. There was a weak correlation of PACAP levels with Migraine Disability Assessment score. PACAP levels also correlated with total headache days (48).

A recent study investigated the role of serum biomarkers, including PACAP, in the development and transformation of a migraine. Chronic migraine, episodic migraine and healthy, headache–free volunteers were compared. Their results were the opposite to the findings of the aforementioned study. Serum PACAP levels in episodic migraineurs were significantly higher than those of the control group. No significant changes were detected between chronic and episodic migraine group and chronic migraine and healthy control group (49).

A recent study examined the effects of an 8–week–long regular moderate– and high–intensity aerobic training on migraine headache indices and on serum biomarkers including PACAP in non–athlete female migraineurs. The findings showed that after 8 weeks of both moderate– and high–intensity aerobic training, the intensity, the duration and the frequency of the migraine headaches were reduced. However, neither training protocols had an effect on serum levels of PACAP (50).

#### 2.4.2 Episodic cluster headache

In a pilot study, episodic cluster headache (ECH) patients were compared to age–matched healthy controls. In the inter–bout period of ECH significantly lower plasma PACAP levels were detected than in the control group. However, PACAP levels were significantly elevated in the plasma during ECH attacks in comparison to the inter–bout phase in the same patients (51).

In a randomized, double–blind, placebo–controlled, two–way cross–over study, patients with episodic cluster headache in an active phase, episodic cluster headache patients in remission and patients with chronic cluster headache were investigated interictally and during experimentally induced cluster headache attacks. Significantly higher baseline PACAP levels were detected in episodic cluster headache patients in active phase, compared to chronic cluster headache patients. There was no significant change in plasma PACAP levels in case of calcitonin gene–related peptide–induced cluster headache attacks (52).

#### 2.4.3 Pituitary adenoma-associated headache

In case of pituitary adenoma–associated headache, significantly higher plasma PACAP levels were found compared to pituitary adenoma patients without headache 72 hours pre– and post–operatively (pituitary adenoma resection). Plasma PACAP levels remained high in patients who only showed little improvement in their headache compared to those who had significant improvement 72 h after the operation (53).

### 2.5 Depression and anxiety disorders

Geriatric depression and anxiety disorders are highly important health problems in aging population (54–57). Evidence from animal models and human studies suggests that PACAP is involved in the development of diverse psychiatric disorders as it plays an important role in regulating stress effects and stressful situations, this regulation being exerted *via* a change in the peptide levels (58). Regarding generalized anxiety disorder (GAD), a preliminary study was published in 2020. The authors found, for the first time, that GAD was associated with lower concentrations of circulating plasma PACAP in females compared to non–psychiatric controls (59).

### 2.6 Post–traumatic stress disorder

A randomized, sham–controlled, double blind pilot study examined the effects of transcutaneous cervical vagus nerve stimulation (tcVNS) on PACAP levels in a three–day chronic stress laboratory paradigm involving serial traumatic and mental stress exposures in healthy individuals with a history of exposure to psychological trauma and patients with post–traumatic stress disorder (PTSD). Although the experiment was designed to examine the therapeutical potential of tcVNS in neuro–biological stress–responses, PACAP served as a dynamic and objective biochemical marker that could measure stress severity. The authors found that acute traumatic and mental stressors are associated with increased PACAP levels in the peripheral blood in traumatized individuals both with and without PTSD. They also concluded that longitudinal monitoring of PACAP levels may potentially be useful to follow personalized, adaptive dosing strategies or to identify respondent and non–respondent patients (60). This is in accordance with the results of the pioneer study conducted by Ressler et al., where PTSD symptoms were significantly positively correlated with plasma PACAP levels in females (61).

## 3 Cardiovascular and cerebrovascular diseases

### 3.1 Ischemic heart disease and heart failure

Aging is an important risk factor for ischemic heart disease and heart failure (62–64). Previous studies have identified diverse cell autonomous and nonautonomus pathways involved in regulation of cardiac and vascular aging processes (65–69). *In vitro* and *in vivo* studies, focusing on protective effects of PACAP against ischemic events, confirmed that PACAP confers significant anti–aging effects in the cardiovascular system (70). PACAP increased cell viability and decreased apoptosis in cultured cardiomyocytes exposed to ischemia/reperfusion. Furthermore, PACAP treatment could increase cell survival and decrease cell death in cardiomyocytes exposed to short preconditioning ischemia followed by ischemia/reperfusion. Regarding *in vivo* data, PACAP mRNA expression increased in mice following acute myocardial infarction. PACAP38 immunoreactivity was high three days after the infarction, indicating that it plays an important role in remodeling. PACAP–deficient mice suffered significantly more severe DNA damage and apoptotic cell death in doxorubicine–induced cardiomyopathy than wild types. PACAP treatment proved to be protective against mitoxantrone induced cardiotoxicity and radiation–induced heart disease (70, 71).

In a study published in 2019, alterations of blood PACAP levels were measured in patients with chronic heart failure caused by primary dilated cardiomyopathy and ischemic cardiomyopathy. The relationship between serum levels of PACAP and other reliable biomarkers of heart failure (HF) was also examined. In patients suffering from mild HF caused by chronic ischemia, a significant strong negative correlation and a linear relationship was detected between PACAP and N–terminal prohormone of brain natriuretic peptide (NT–proBNP) levels. In moderate HF, the authors found a significant moderate negative correlation between PACAP and NT–proBNP levels only in the ischemic subgroup. A positive tendency with a weak positive correlation was shown between serum PACAP and systolic left ventricular function only in case of ischemic cardiomyopathy (71).

In a recently published study, alteration of PACAP levels in acute and chronic HF were examined as well as the correlations between PACAP and HF predictors. In acute HF significantly higher plasma PACAP levels were detected compared to the chronic group and healthy controls. Moreover, patients with chronic HF had significantly lower PACAP levels compared to both acute HF and the control group. A weak significant negative correlation was found between NT–proBNP and PACAP levels in chronic HF patients. Taking the etiology of cardiomyopathy into account, a positive connection was found between PACAP and NT–proBNP levels in acute cases and significantly remarkable strong negative correlation was detected between the two examined markers in chronic HF group. In the merged HF patient group a significant weak positive correlation was observed between C–reactive protein (CRP) and PACAP. Moreover, taking the type of HF into account, a significant strong positive correlation was detected between the aforementioned markers. Regarding pro– and anti–inflammatory cytokines, interleukin (IL) 1β, IL–2, IL–4 levels were significantly lower in the chronic HF group compared to both acute HF and healthy groups, while IL–10 levels were significantly higher in acute HF patients compared to the controls but not to the chronic HF group. Each abovementioned IL showed significant positive correlation to PACAP concentrations in HF cohort. More remarkable positive correlation was detected between these cytokines and plasma PACAP levels taking the type of HF into consideration (72).

A recent translational study investigated plasma PACAP levels in patients with ST–segment elevation myocardial infarction (STEMI). Significantly higher plasma PACAP levels of STEMI patients were observed before percutaneous coronary intervention (PCI) and a significant decrease was detected right after PCI. This decreasing tendency was also present 4, 24 and 48 h after PCI. Significantly higher PACAP levels were found in the 0 h samples of the STEMI patients compared to the controls. In contrast, significantly higher plasma PACAP levels were detected in the control group compared to STEMI patients 48 h after PCI. A significant weak negative correlation was found between all the time–matched plasma PACAP and cardiac troponin levels in the STEMI patient group (73).

### 3.2 Stroke and cerebromicrovascular diseases

Aging is a major risk factor for vascular cognitive impairment (VCI), the second most common form of cognitive impairment after AD. A wide array of functional and structural alterations of the cerebral microvasculature were shown to contribute to the pathogenesis of VCI (74–77) including cerebromicrovascular rarefaction (5, 78) and a related decline in cerebral blood flow (79, 80), microhemorrhages (81–84), microinfarcts (85, 86), impaired glymphatics function (87), blood brain barrier disruption (88, 89), consequential neuroinflammation (90), impaired neurovascular coupling (91–93) systemic endothelial dysfunction (4, 94) and endothelial senescence (95). Stroke is an important consequence of cerebrovascular aging. There is increasing evidence that various circulating factors play a key role in regulation of vascular aging (96, 97). Among them, PACAP seems to have an important role in modulation of endothelial function, angiogenesis and cerebrovascular health. PACAP has been reported to be neuroprotective in several neuronal cultures against various toxic insults, as well as in *in vivo* models of different neuronal injuries including global ischemia, and both transient and permanent focal ischemia. *In vivo* studies found that PACAP reduced apoptosis and the inflammatory response reaction in the ischemic penumbra. Furthermore, PACAP induced neuronal protection *via* receptor and receptor independent ways. PACAP treatment was able to improve functional deficits in stroke animal models (70, 98).

In a study conducted with high number of spontaneous (non–traumatic) intracerebral hemorrhage patients, higher plasma PACAP were found than in age– and sex–matched healthy controls. Moreover, plasma PACAP levels were higher in non–survivors than in survivors. Multivariate analysis emerged PACAP plasma concentration as an independent predictor for 1–week, 6–month mortality and 6–month overall survival (98). A study involving 118 non–traumatic aneurysmal subarachnoid hemorrhage patient and healthy controls revealed similar results, as admission PACAP levels were significantly elevated in patients compared to healthy controls. PACAP levels were also associated with the clinical severity. PACAP levels proved to be an independent predictor for 6–month mortality and 6–month unfavorable outcome and 6–month overall survival (99).

## 4 Diseases of the gastrointestinal tract and the kidney

Aging is associated with the decline in gastrointestinal function and the severity and poor prognosis of various liver (100, 101) or kidney diseases (102). Several studies have shown the involvement of PACAP in the gastrointestinal and urogenital tract in physiological and pathological processes, including inflammatory disorders, diabetes, intoxications and neoplastic processes (103, 104). The nephroprotective effect of both exogenous and endogenous PACAP was revealed by numerous *in vitro* experiments in case of oxidative stress and hypoxia, drug induced nephropathies, diabetic nephropathy and myeloma kidney injury. Data from *in vivo* studies proved that not only PACAP treatment, but also the endogenously present peptide is able to exert protective effects in ischemia/reperfusion, diabetic nephropathy, myeloma kidney injury, renal amyloidosis and in different type of drug induced kidney pathologies, including nephrotoxic antibiotics, chemotherapeutics or contrast agents (70, 103).

In nephrotic syndrome patients, parallel loss of urine PACAP and decreased plasma PACAP levels was detected. Interestingly, after bilateral nephrectomy, plasma PACAP levels normalized in parallel with the normalization of elevated platelet counts (105). An earlier human study showed decreased PACAP levels in chronic hepatitis B patients compared to healthy controls, which diminished after antiviral therapy (106).

Regarding hepatic diseases, the expression of PACAP and its receptors was increased in liver ischemia/reperfusion injury model, whereas the hepatocellular damage was exacerbated in mice lacking PACAP. Furthermore, PACAP treatment had been found protective against hepatic cell death and inflammatory response. In obesity–related hepatic pathology PACAP enhanced glucose and lipid metabolism and thus protected against inflammation and steatosis (70, 107).

Significant decrease of plasma PACAP levels was found in patients with liver cirrhosis compared to healthy controls. Furthermore, a progressive decrease was also observed in the different Child–Pugh stages of the disease. Significantly lower plasma PACAP levels were present in patients with Child–Pugh stage C compared to those with stages A and B. A markedly decreased plasma PACAP concentration was detected in cirrhosis with Child–Pugh B stage compared with A stage. ROC curve analysis demonstrated that low plasma PACAP level may act as an indicator for disease progression of liver cirrhosis determined by Child–Pugh classification. PACAP levels were significantly and negatively associated with the liver histology severity score and with the alanine aminotransferase and aspartate aminotransferase levels. Plasma PACAP concentrations showed positive correlation with the nutritional status in liver cirrhosis (107).

## 5 Diseases of the musculoskeletal system

Age–related diseases of the musculoskeletal system are important determinants of quality of life in old age. Our current understanding is that cell autonomus and nonautonomus pathways interact to regulate aging processes in the bone, joints and skeletal muscle, which are also modulated by lifestyle factors (108–112). PACAP had a positive effect on chondrogenesis and bone formation in both *in vivo* and *in vitro* studies. Furthermore, because PACAP is required for proper bone architecture and callus formation, lacking of endogenous PACAP results in morphological and biochemical changes in articular cartilage, potentially making this tissue more susceptible to degenerative disorders. Based on animal experiments PACAP appears to maintain the equilibrium between matrix formation and matrix breakdown, which is crucial for optimal cartilage matrix production in young animals. The expression of the PAC1 receptor was also changed in specific layers of cartilage in osteoarthritis and decreased during oxidative stress (7, 113, 114). As PACAP regulates critical processes related to the maintenance of the structural and functional integrity, development and regeneration of skeletal elements, therefore, dysregulation of PACAP signaling in degenerative cartilage diseases, arthritis and osteoporosis is receiving increasing research attention.

A study by Sun et al. (114) examined the association of serum and synovial fluid (SF) PACAP concentrations in primary knee osteoarthrosis (PKOA). In PKOA patients significantly lower PACAP concentrations were detected in SF compared to controls, while there was no significant difference between the serum PACAP levels. SF PACAP levels were negatively correlated with self–reported pain. In contrast, positive correlation was observed in functional severity, like Oxford Knee Score, American Knee Society knee score and American Knee Society knee function score. Regarding the radiological severity, SF PACAP levels were negatively correlated with Kellgren–Lawrence (K–L) grades, inflammatory cytokine IL–1β and cartilage damage marker matrix metalloproteinase–3 (MMP–3). Based on the ROC curve analysis the authors concluded that decreased PACAP expression along with up–regulation of MMP–3 in SF during PKOA might act as two potential markers for the K–L grade at different stages. The effect of knee joint hyaluronic acid (HA) injection on SF PACAP levels was also examined. After HA injection SF PACAP levels were significantly elevated at the 8th week compared to the baseline and the 4th week (114).

The biomarker potential of PACAP in post–traumatic knee osteoarthritis (PTKOA) following anterior cruciate ligament (ACL) lesion was investigated by Sun et al. (115). ACL injury patients were compared to controls. Similar to PKOA, significantly lower SF PACAP concentrations were measured compared to controls, while there was no significant difference between the serum PACAP levels. SF PACAP levels were negatively related to the degree of meniscus injury assessed by MR imaging grading system. Negative correlation was found between SF PACAP levels and visual analogue scale used to monitor the pain. On the other hand, SF PACAP levels were positively associated with functional ability assessed by Lysholm score and International Knee Documentation Committee score. Significant negative correlation was observed between SF PACAP levels and modified Mankin’s histological grading system used to investigate the histological and pathological changes of lesioned cartilage obtained from PTKOA patients. SF PACAP levels were negatively related to synovial fluid expressions of IL–1β and TNF–α. Based on ROC curve analysis PACAP may serve as a favorable indicator for later stage of meniscus injury than IL–1β and TNF–α (115).

A recent study examined the changes of serum PACAP levels in case of patients with non–traumatic osteonecrosis of femoral head (ONFH) and healthy controls. Serum PACAP levels were significantly lower in non–traumatic ONFH patients compared to the levels observed in healthy controls. Regarding radiological severity, serum PACAP levels were inversely related to Association Research Circulation Osseous classification stage. Positive correlation was found between serum PACAP levels and the Harris Hip Score. In contrast, the symptomatic severity measured by visual analogue scale showed negative correlation with serum PACAP levels. Regarding existing biochemical markers, serum PACAP concentrations were found to be negatively correlated with serum IL–33 levels and positively associated with β C–terminal telopeptide of type I collagen levels. The results suggested that reduced serum PACAP concentrations may serve as an early diagnostic marker to evaluate disease development of non–traumatic ONFH (116).

Significantly lower serum PACAP levels were observed in post–menopausal osteoporosis (PMOP) compared to age–matched healthy controls. Serum levels of PACAP in vertebrae fracture patients in PMOP were significantly lower than that in non–fracture PMOP patients. Moreover, serum PACAP levels were significantly negatively correlated to Genant radiological grade of vertebral fractures. Significant positive correlation was found between serum PACAP concentrations and bone mineral density at left femoral neck, total hip and L1–L4 lumbar spine. Serum PACAP levels were negatively associated with numerical rating scale for pain. In addition, significant positive correlations were identified between serum PACAP levels and 6–min walking and sit–to–stand test. N–terminal propeptide of type I procollagen, a marker of bone formation, correlated positively with serum PACAP levels, while C–telopeptide of type I collagen, a marker of bone resorption, showed negative correlation with serum PACAP concentrations (117).

## 6 Critical illness

As the population increased globally, an increasingly older polytrauma population has been observed. Nowadays 23% of all trauma admissions involving patients over 65 years, and trauma associated death is the fifth leading cause of death in the elderly. Aging is associated with decreased physical reserve, and the immune response is slower and less intense, leading to increased infection and wound complication rates, therefore mortality rates in the geriatric population are increased, even when controlling for existing comorbidities (118–121). The sympathetic nervous system and the hypothalamo–hypophyseal–adrenal axis are unquestionably involved in different critical diseases. Furthermore, there is bidirectional cross–talk between the neuroendocrine and immunological systems *via* several mediators. PACAP, as the master regulator of stress adaptation, was also found to have immunomodulatory properties *in vivo* in reducing the harmful effects of septic shock by balancing pro– and anti–inflammatory elements (113, 122, 123). A study examined the alteration of plasma and CSF levels of PACAP in severe traumatic brain injury (TBI). Higher CSF and plasma PACAP levels were measured in TBI patents compared to the controls. Furthermore, both plasma and CSF levels are increased within the first two days after the injury. Interestingly, in surviving patients, plasma and CSF levels were parallel, while in patient, who died within the first week CSF levels were almost half compared to plasma levels. Nether plasma, nor CSF PACAP levels showed no correlation with other clinical parameters (122).

Monitoring the dynamic balance of pro– and anti–inflammatory processes for early detection of infectious complications in case of polytraumatic patients is obligatory in modern polytrauma care. PACAP has well–known immunomodulatory and anti–inflammatory effects and plays an important role in stress adaptation, therefore, a recent study examined the biomarker potential of PACAP in early post–traumatic period in cases without septic complications. Serum PACAP levels of polytrauma patients were measured and compared to other conventional and non–conventional laboratory markers. Changes of PACAP levels showed no statistical significance, but moderately increasing PACAP levels were detected with a peak on day 4 and slightly decreased thereafter on day 5. Between serum PACAP and CRP levels a significant weak positive correlation was found on day 4 and a significant moderate positive correlation on day 5. The authors explain this parallel evolution of serum concentrations by an endogenous response to the trauma–induced systemic inflammatory response syndrome, as PACAP exerts its anti–inflammatory effects. Examining all five days together, a statistically significant correlation was detected between PACAP and leukocyte anti–sedimentation rate levels (123).

Elderly with Coronavirus disease 2019 suffered more severe cases and complications therefore this population showed increased morbidity and mortality (124–126). A recent study examined the possible protective and biomarker role of PACAP and VIP during SARS‐CoV‐2 infection, but no significant differences were found between PACAP plasma levels and the groups analyzed, inflammatory markers, viral load (127).

## 7 Conclusions and perspectives

**Figure 2:**
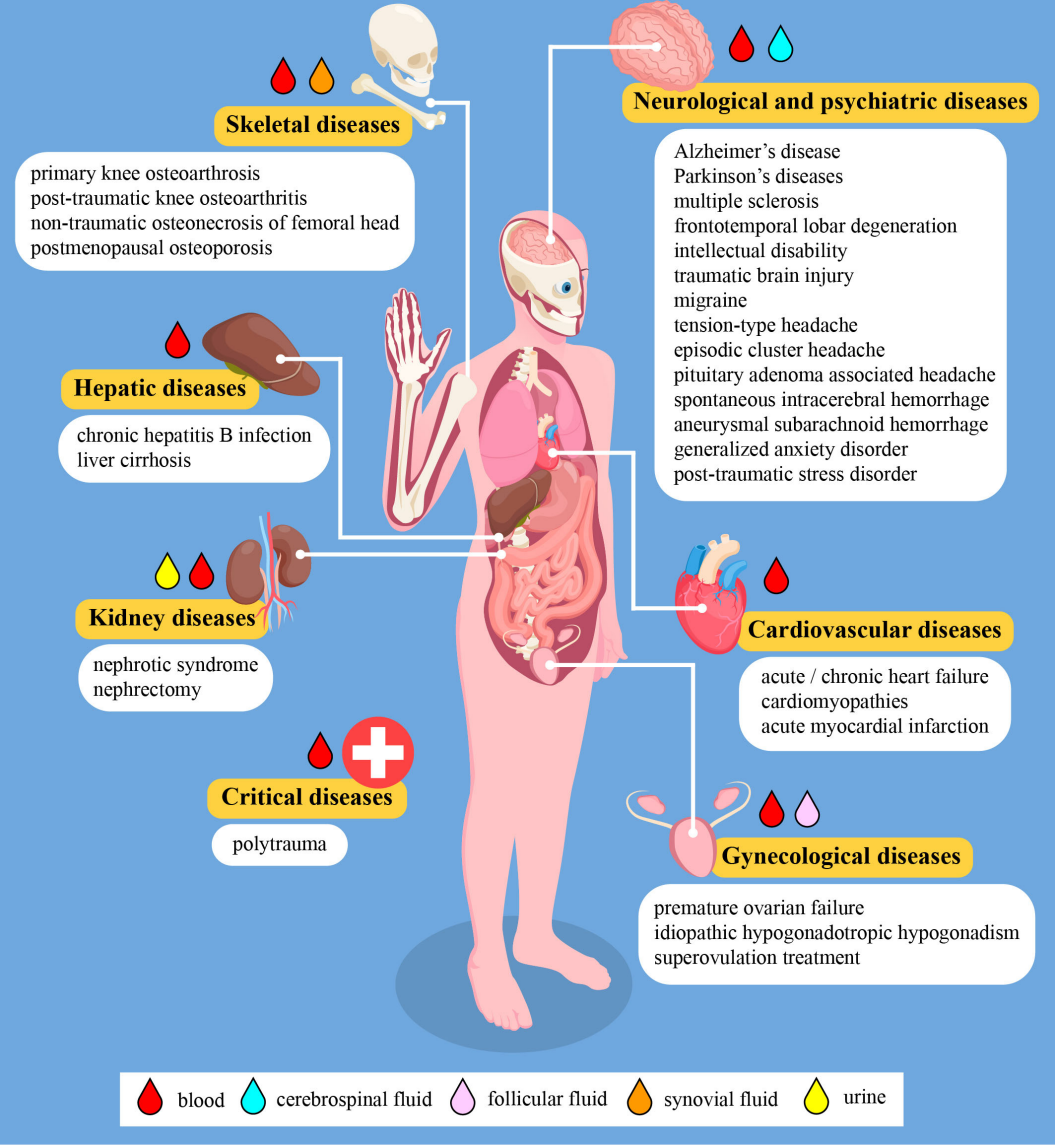
Schematic figure about human diseases where changes of the PACAP levels were investigated. The figure contains modified elements designed by macrovector/Freepik (https://www.freepik.com).

Aging is an unavoidable time–dependent deterioration of body functions, which is thereby associated with a higher prevalence of chronic diseases and conditions (128). Developing and evaluating biological, radiological, and other biomarkers can help us to measure the well–being of the elderly population as well as symptoms of disease and disability which may lead to declines in overall health and quality of life (129). *In vivo* and *in vitro* data showed that PACAP tissue levels and receptor expression alter with aging, as do PACAP–mediated actions and signaling pathways, therefore PACAPerg signaling may be crucial in the development of age–related diseases. Mice without endogenous PACAP exhibit several metabolic, behavioral, and inflammatory changes. In addition, PACAP–deficient mice display increased susceptibility to a variety of stressors under pathological conditions, some of which are also present during physiological or pathological aging. The lack of endogenous PACAP accelerate age–related degeneration and PACAP knockout animals exhibit age–related degenerative signs earlier, so PACAP deficiency can be used nowadays as a model of aging due to the decreased anti–apoptotic, anti–inflammatory, and antioxidant activities (6, 7, 10, 17, 27, 113). Although we are at the beginning of a long journey, the increasing number of clinical data from the past years suggest that PACAP has high translational potential as a diagnostic and prognostic neuro–biomarker in a wide range of diseases, including age–related diseases. **Figure 2** summarizes all human diseases where changes of PACAP levels were investigated and the presently reviewed results are summarized in **Table 1**. There are numerous questions waiting to be answered, including the exact source of PACAP in different body fluids or the mechanisms contributing to alteration of PACAP levels. There are also several limitations at the moment, like the usually small sample size, comparability of study results and methodological difficulties. Our current knowledge is limited to only a few body fluids (mostly to blood) and physiological and pathological processes, which we are aspiring to broaden. The majority of the early studies in this field focused on the neurological diseases including neurodegenerative disorders and headaches. Evidently, this area of research will continue to extend in the future. On the other hand, new research fields have appeared in the past five years with encouraging results. The biomarker potential of PACAP in case of bone and joint disorders or in cardiology, as well as in intensive therapy seems to be a promising topic, especially in combination with other biomarkers or biomarker panels. We believe that the growing body of evidence regarding the biomarker value of PACAP could serve as a good source for multi–center clinical trials.

**Table 1:** Changes in PACAP levels in human pathological conditions.

Condition or disease	Patient number	Sample, method	Level ranges	Change of PACAP	Reference
Neurological and psychiatric diseases					
Alzheimer’s disease	9 (vs. 7 controls)	CSF, ELISA	1.8 vs. 2.1 ng/mL	↓ (vs. controls),	(26)
				correlated with DRS–R	
Alzheimer’s–related cognition decline spectrum	16 AD (vs. 9 MCI–AD vs. 10 CN)	CSF, ELISA	1.61 vs. 1.80 vs. 1.61 ng/mL	↓ (vs. MCI–AD),	(28)
				↓ (vs. CN),	
				correlated with DRS–R	
Parkinson’s disease with dementia	8 (vs. 7 controls)	CSF, ELISA	2.1 ng/mL in both	no change	(26)
Parkinson’s disease	72 (vs. 71 controls)	serum, ELISA	76.02 ± 43.78 vs. 154.96 ± 76.54 pg/mL	↓ (vs. controls),	(33)
				negative correlation with attention/memory	
Parkinson’s disease	106 (vs. 37 controls)	plasma, ELISA	normalized data only	↓ (vs. controls),	(34)
				↑ in patients with DBS therapy vs. without DBS therapy,	
				correlation with PD subtypes and clinical stages	
multiple sclerosis	145 (vs. 73 controls)	serum, ELISA	57,179.11 vs. 61,419.05 fg/mL,	no change (vs. controls),	(37)
			female patients 62,466.400 vs. male patients 48,516.214 fg/mL	↑ in female vs. male patients	
multiple sclerosis	20 (vs. 27 controls)	plasma and CSF, ELISA	plasma: 4.20 ± 3.03 vs. 4.23 ± 2.64 pg/mL	no change in plasma	(38)
			CSF: 4.15 ± 1.23 vs. 6.72 ± 3.12 pg/mL	↓ (vs. controls) in CSF	
migraine	38 (vs. 20 controls)	plasma, ELISA	interictal: 1929 ± 706 and ictal: 1638 ± 738 vs. 830 ± 275 pg/mL	↑ (vs. controls),	(43)
				no change ictal vs. interictal,	
				negative correlation with attack frequency per month	
migraine	87 (vs. 40 controls)	plasma, RIA	interictal: 24.60 ± 3.59 and ictal: 27.39 ± 4.67 vs. 26.54 ± 4.43 fmol/mL	↓ interictal vs. controls	(44)
				↓ interictal and ↑ ictal	
migraine with sumatriptan treatment	15	plasma, RIA	interictal: 21 ± 3 vs. ictal: 36 ± 3 vs. 1 h after treatment: 26 ± 3 pmol/L	↓ interictal and ↑ ictal	(45)
				↓ after treatment	
migraine	26	plasma, RIA	normalized data only	correlation with alterations of the white matter microstructure detected by diffusion MRI	(46)
chronic vs. episodic migraine	86 vs. 35 (vs. 32 controls)	serum, ELISA	109.8 ± 43.8 vs. 98.8 ± 34.3 vs. 108.7 ± 43.0 pg/mL	no change (vs. controls)	(47)
chronic vs. episodic migraine	101 vs. 98 (vs. 97 controls)	serum, ELISA	204.931 (101.08–597.64) vs. 94.992 (65.77–128.48) vs. 103.142 (59.42–123.97) pg/mL [median and (IQ)]	↑ in chronic migraine (vs. episodic and controls)	(48)
chronic vs. episodic migraine	36 vs. 23 (vs. 30 controls)	serum, ELISA	2.72 (1.06) vs. 2.73 (0.46) vs. 2.57 (0.64) ng/mL [median and (IQR)]	↑ in episodic migraine vs. controls,	(49)
				no change in chronic migraine vs. controls	
effect of aerobic training on migraineurs	15 + 15 (vs. 15 controls)	plasma, ELISA	normalized data only	no change (vs. controls)	(50)
episodic cluster headache	9 (vs. 9 controls)	plasma, RIA	inter–bout: 24.4 (2.68) vs. 30.5 (8.84) fmol/mL, ictal: 28.8 (3.40) vs. 30.5 (8.84) fmol/mL [median and (IQR)]	↓ in inter–bout period,	(51)
				↑ in attack period	
episodic vs. chronic cluster headache	18 vs. 13	plasma, RIA	4.0 ± 0.8 vs. 3.3 ± 0.7 pmol/L	↑ in episodic cluster headache patients in active phase	(52)
headache associated with vs. without pituitary adenoma	33 vs. 30	plasma, ELISA	23.32 ± 6.51 vs. 20.51 ± 4.73 pmol/L	↑ in headache associated with pituitary adenoma,	(53)
				PACAP concentration remained high in patients who had little improvement in headache	
generalized anxiety disorder	88 (vs. 110 controls)	plasma, RIA	normalized data only	↓ in females only	(59)
effect of acute stress	10 (vs. 20 controls)	blood, ELISA	normalized data only	↑ both groups	(60)
post–traumatic stress disorder	64	plasma, RIA	low: < 20 pM,	↑ in females only	(61)
			high: > 20 pM	correlated with symptoms in females only	
Cardiovascular and cerebrovascular diseases					
heart failure in primary dilated vs. ischemic cardiomyopathy	9 vs. 33	plasma, RIA	normalized data only	↓ in HF with ischemic background,	(71)
				negative correlation with NT–proBNP in ischemic group,	
				positive correlation with systolic left ventricular function in ischemic group	
acute vs. chronic heart failure	13 vs. 33 (vs. 13 controls)	plasma, ELISA	normalized data only	↑ in acute HF,	(72)
				↓ in chronic HF,	
				negative correlation with NT–proBNP in case of chronic HF,	
				positive correlation with CRP in merged group,	
				positive correlation with low cytokine levels in case of chronic HF	
ST–segment elevation myocardial infarction	16 (vs. 12 controls)	plasma, ELISA	normalized data only	↑ in STEMI before intervention,	(73)
				↓ in STEMI after intervention,	
				negative correlation with cardiac troponin in patient group	
non–traumatic basal ganglia hemorrhage	150 (vs. 150 controls)	plasma, ELISA	268.0 ± 112.6 vs. 72.0 ± 24.2 pg/mL	↑ (vs. controls),	(98)
				positive correlation with NIHSS scores and hematoma volume,	
				independent predictor for mortality	
non–traumatic aneurysmal subarachnoid hemorrhage	118 (vs. 118 controls)	plasma, ELISA	296.6 ± 119.7 vs. 77.1 ± 17.9 pg/mL	↑ (vs. controls),	(99)
				positive correlation with WFNS and Fisher score,	
				independent predictor for mortality	
Diseases of the gastrointestinal tract and the kidney					
nephrotic syndrome	28 (vs.10 controls)	plasma and urine, WB	normalized data only	↓ in plasma (vs. controls),	(105)
				↑ in urine (vs. controls),	
				↑ after nephrectomy	
chronic hepatitis B with lamivudine therapy	25 (vs. 25 controls)	plasma, RIA	before treatment 22.62 ± 8.27, after treatment: 49.6 ± 16.45 vs. 65.18 ± 12.61 pg/mL	↓ (vs. controls),	(106)
				↑ after treatment	
liver cirrhosis	113 (vs. 110 controls)	plasma, ELISA	306.1 ± 55.1 vs. 385.5 ± 48.9 mg/dL	↓ (vs. controls),	(107)
				negative correlation with clinical stages	
Diseases of the musculoskeletal system					
primary knee osteoarthrosis	101 (vs. 62 controls)	serum and SF, ELISA	serum: 93.2 ± 12.5 pg/mL vs. 94.9 ± 15.5 pg/mL,	no change (vs. controls) in serum,	(114)
				↓ in SF,	
			SF: 239.5 ± 36.4 vs. 305.1 ± 12.5 pg/mL	↑ in SF after HA injection,	
				negative correlation with radiological stages and clinical severity (SF)	
post–traumatic knee osteoarthrosis	72 (vs. 60 controls)	serum and SF, ELISA	serum: 86.0 ± 15.3 pg/mL vs. 82.8 ± 19.7 pg/mL,	no change (vs. controls) in serum,	(115)
				↓ in SF,	
			SF: 202.0 ± 48.3 vs. 326.0 ± 66.7 pg/mL	negative correlation with radiological stages and clinical severity	
non–traumatic osteonecrosis of femoral head	102 (vs. 95 controls)	serum, ELISA	212.8 ± 24.4 vs. 303.7 ± 19.7 pg/mL	↓ (vs. controls),	(116)
				negative correlation with radiological stages and clinical severity	
postmenopausal osteoporosis	123 (vs. 120 controls)	serum, ELISA	55.7 ± 21.3 vs. 91.5 ± 19.5 pg/mL	↓ (vs. controls),	(117)
				negative correlation with radiological stages,	
				positive correlation with bone mineral density, negative correlation with clinical severity	
Critical illness					
traumatic brain injury	38 (vs. 14 controls)	plasma and CSF, RIA	plasma: 37.458 ± 12.463 vs. 14.514 ± 3.080 fmol/mL	↑ in plasma and CSF (vs. controls),	(122)
			CSF: 28.978 ± 10.116 vs. 16.090 ± 1.018 fmol/mL	correlation with mortality	
polytrauma	13	plasma, ELISA	normalized data only	↑ days 1–4 (tendency only),	(123)
				↓ day 5 (tendency only),	
				positive correlation with CRP on day 4 and 5.	
coronavirus disease 2019	24	plasma, ELISA	no data	no change	(124)

PACAP, pituitary adenylate cyclase activating polypeptide; ↑, increase; ↓, decrease; CSF, cerebrospinal fluid; ELISA, enzyme–linked immunosorbent assay; DRS–R, Mattis Dementia Rating Scale–Revised; AD, Alzheimer’s disease; MCI–AD, mild cognitive impairment in Alzheimer’s disease; CN, normal cognitive; DBS, deep brain stimulation; PD, Parkinson’s disease; RIA, radioimmunoassay; MRI, magnetic resonance imaging; IQR, interquartile range; HF, heart failure; NT–proBNP, N–terminal prohormone of brain natriuretic peptide; CRP, C–reactive protein; STEMI, ST–segment elevation myocardial infarction; NIHSS, National Institutes of Health Stroke Scale; WFNS, World Federation of Neurosurgical Societies; WB, Western blot; SF, synovial fluid; HA, hyaluronic acid.

## Author contributions

DT and DR, AT conceptualized the paper. DT and LS constructed the table. DT, DR, AT and LS wrote parts of the review. All authors contributed to the article and approved the submitted version.
